# Supplementary material for: Investigation of the effectiveness of no-reference metric in image evaluation in nuclear medicine
Source: PLoS One. 2024 Nov 21;19(11):e0310305. doi: 10.1371/journal.pone.0310305 (PMC11581283; doi:10.1371/journal.pone.0310305)
Supplement: Attachment — Submitted filename: Comments to the author_ Plose one.docx [file pone.0310305.s001.docx]

This paper examines whether No-Reference metrics can be applied to image quality evaluations for clinical images in nuclear medicine. To this end, 14 images with different numbers of pixels and acquisition times were created and extracted from Biograph Vision. The paper is well organized but not well written. I have some minor points that need to be addressed.

1. The contribution is not clear and needs to be highlighted.
2. The introduction should be modified so that the last paragraphs should be divided as paper contributions and finds and paper organization as a separated subsections.
3. Please make the contributions as a bullet points.
4. The novelty of the paper needs to be modified to be more clear.
5. Some grammar errors are discovered so please try to proofread the paper again.
6. I would suggest make it clear what have been done in comparison to the state of the art methods showing the contribution of the paper.
7. I would suggest adding equation number.
8. I have found that there is an evaluation before the results section. I would suggest making the evaluation in results section part to make it clear to the reader.
9. The author state that (The total score in shown in bottom row of..) what dose that means please correct.
10. Tables should not be splatted into two pages please correct table 14.
11. There are many references in the discussion part after the results. Why not stated these references in the introduction part.
12. The conclusion is too short and not clear please modify.
