# Supplementary material for: Investigation of the effectiveness of no-reference metric in image evaluation in nuclear medicine
Source: PLoS One. 2024 Nov 21;19(11):e0310305. doi: 10.1371/journal.pone.0310305 (PMC11581283; doi:10.1371/journal.pone.0310305)
Supplement: Attachment — Submitted filename: 20240329Journal Requirements and review comments.docx [file pone.0310305.s002.docx]

Thank you for your careful review and your comments.

We have made changes to comply with your suggestions. The revised parts of the manuscript are written in red.

Journal Requirements:

1 When submitting your revision, we need you to address these additional requirements.

→We have made the changes according to your suggestions.

2 Note from Emily Chenette, Editor in Chief of PLOS ONE, and Iain Hrynaszkiewicz, Director of Open Research Solutions at PLOS: Did you know that depositing data in a repository is associated with up to a 25% citation advantage (https://doi.org/10.1371/journal.pone.0230416)? If you’ve not already done so, consider depositing your raw data in a repository to ensure your work is read, appreciated and cited by the largest possible audience.

→ We have read and understood the contents.

3 Please note that PLOS ONE has specific guidelines on code sharing for submissions in which author-generated code underpins the findings in the manuscript. In these cases, all author-generated code must be made available without restrictions upon publication of the work.

→ We have read and understood the contents.

4 PLOS requires an ORCID iD for the corresponding author in Editorial Manager on papers submitted after December 6th, 2016.

→ The authors and corresponding author (Shigeaki Higashiyama) are registered ORCID members.

5 We note that your Data Availability Statement is currently as follows: [All relevant data are within the manuscript and its Supporting Information files.]

→ All relevant data are present in the manuscript and Supporting Information files.

6 We note that Figure 1 and 4 in your submission contain copyrighted images. All PLOS content is published under the Creative Commons Attribution License (CC BY 4.0), which means that the manuscript, images, and Supporting Information files will be freely available online, and any third party is permitted to access, download, copy, distribute, and use these materials in any way, even commercially, with proper attribution. For more information, see our copyright guidelines: http://journals.plos.org/plosone/s/licenses-and-copyright.　We require you to either (1) present written permission from the copyright holder to publish these figures specifically under the CC BY 4.0 license, or (2) remove the figures from your submission:

→ Figures 1 and 4 are images that I created and used in this paper. This is not a quotation from any literature or book. I had inquired about permission to use the preprint without peer review, but I received a reply stating that there was no need to confirm the license. I have added that email as a PDF attachment to the other file.

Comments to the Author

1-3 and 6 → We do not require a response.

4→ We utilized a paid English proofreading of the main text. Certificate of editing was attached the file as PDF.

5 Review Comments to the Author

To Reviewer #1: (No Response)

To Reviewer #2

1、	The contribution is not clear and needs to be highlighted.

→ We have modified the introduction to include a subsection specifically dedicated to outlining the contributions and findings of the paper.

2、The introduction should be modified so that the last paragraphs should be divided as paper contributions and finds and paper organization as a separated subsections.

→ We divided the Introduction into paragraphs on Contributions and findings.

3 Please make the contributions as a bullet points.

→ We divided the Introduction and Abstract into paragraphs and clearly stated the novelty.

4、The novelty of the paper needs to be modified to be more clear.

→ A sentence explaining the novelty of this research has been added to the introduction.

5、Some grammar errors are discovered so please try to proofread the paper again.

→ We have used a paid English proofreading service. Certificate of editing was attached the file as PDF.

6、 I would suggest make it clear what have been done in comparison to the state of the art methods showing the contribution of the paper.

→ In the introduction, we have added a description on the novel aspects of this study compared to what has been presented in past papers.

7、 I would suggest adding equation number.

→ We have numbered the equations in the text.

8、 I have found that there is an evaluation before the results section. I would suggest making the evaluation in results section part to make it clear to the reader.

→ Based on you suggestion, we have made changes to make the results easier to understand.

9、The author state that (The total score in shown in bottom row of..) what dose that means please correct.

→ We have added a description of the table you pointed out.

10、 Tables should not be splatted into two pages please correct table 14.

→ We have accordingly reorganized Table 14.

11、There are many references in the discussion part after the results. Why not stated these references in the introduction part.

→ Some of the references for the discussion are cited in the methods section.

12、The conclusion is too short and not clear please modify.

→As pointed out, I have further elaborated the content in the conclusion section.
