# Supplementary material for: Investigation of the effectiveness of no-reference metric in image evaluation in nuclear medicine
Source: PLoS One. 2024 Nov 21;19(11):e0310305. doi: 10.1371/journal.pone.0310305 (PMC11581283; doi:10.1371/journal.pone.0310305)
Supplement: Attachment — Submitted filename: Point.docx [file pone.0310305.s003.docx]

**Point-by-point response**

・98: 2 6 MBq basis for inclusion.

・99：PET/CT scannerのBiograph visionは450？600？

・99: The PET/CT scanner used was the Biographic vision 450 or 600?

・112：Pixel size 0.825 mm is only correct for a matrix size of 880 when the transverse FOV is 726 mm.

・113: Is 3 mm correct for Slice thickness instead of 3 cm?

116: Imaging conditions for computed tomography attenuation correction should be stated.

　　The description of Random correction should also be added.

・120: 440 matrix size is more appropriate than 440 pixel size.

：What does 0.4125 mm×0.4125×2 mm^３^mean? If the transverse FOV is 726 mm and the matrix size is 440, the pixel size is 1.65 mm; if the matrix size is 880, the pixel size is 0.825 mm. If the magnification factor is 2x, the pixel size is 0.825 mm for a 440 matrix size and 0.4125 for an 880 matrix size.

・122: Statistical analysis instruments are described, but significance levels are not stated.

・235：CV should be assessed on a uniform phantom. There is also poor evidence for the size and location of the ROI; if CV is measured once in each series, it should be measured at least three times to account for measurement error.

・394: acquisition time is appropriate, not capturing time.

・The matrix size and color scale should be added to Fig 1 and Fig 2 respectively.

・The names of the vertical axes in Fig 6 should be added.

・The names of the vertical and horizontal axes in Figure 8 are mandatory. If the vertical axis is the CV value and the horizontal axis is the acquisition time, they should be described.

・Is the value obtained for the 440 and 880 matrix size for 330 s in the CV results in Table 4 correct?

・The CV values were similar for the 880 matrix size and the 440 matrix size. However, the visual evaluation results show that the 880 matrix size is better for all acquisition time when compared to the 440 matrix size.
