# Supplementary material for: Investigation of the effectiveness of no-reference metric in image evaluation in nuclear medicine
Source: PLoS One. 2024 Nov 21;19(11):e0310305. doi: 10.1371/journal.pone.0310305 (PMC11581283; doi:10.1371/journal.pone.0310305)
Supplement: Attachment — Submitted filename: 20240624Response to Reviewers.docx [file pone.0310305.s004.docx]

Response to Reviewers

1. If the authors have adequately addressed your comments raised in a previous round of review and you feel that this manuscript is now acceptable for publication, you may indicate that here to bypass the “Comments to the Author” section, enter your conflict of interest statement in the “Confidential to Editor” section, and submit your "Accept" recommendation.

Reviewer #1: (No Response) → We have corrected the points raised in the point by point response.

Reviewer #2: All comments have been addressed → Thank you for reviewing the revised manuscript.

2. Is the manuscript technically sound, and do the data support the conclusions?

The manuscript must describe a technically sound piece of scientific research with data that supports the conclusions. Experiments must have been conducted rigorously, with appropriate controls, replication, and sample sizes. The conclusions must be drawn appropriately based on the data presented.

Reviewer #1: Partly　→ Thank you for reviewing the revised manuscript. We have corrected the points raised in the point by point response.

Our study uses phantom data that is accessible from any research institution, not patient clinical data. Therefore, reproducing the methods described in the manuscript is feasible and technically sound. Consequently, we believe that faithfully replicating the methods and evaluation criteria of this paper allows for accurate replication of the conclusions presented in the manuscript. As such, no changes or additions to the evaluation methods or data have been made.

Reviewer #2: Yes　→ Thank you for reviewing the revised manuscript.

3. Has the statistical analysis been performed appropriately and rigorously?

Reviewer #1: I Don't Know　→ We have corrected the points raised in the point by point response.　Our study employed JMP (SAS Japan Co., Ltd) for statistical analysis. Therefore, the statistical analysis in the manuscript is replicable, and technically robust.

Reviewer #2: Yes　→ Thank you for reviewing the revised manuscript.

4. Have the authors made all data underlying the findings in their manuscript fully available?

The [PLOS Data policy](http://www.plosone.org/static/policies.action#sharing) requires authors to make all data underlying the findings described in their manuscript fully available without restriction, with rare exception (please refer to the Data Availability Statement in the manuscript PDF file). The data should be provided as part of the manuscript or its supporting information, or deposited to a public repository. For example, in addition to summary statistics, the data points behind means, medians and variance measures should be available. If there are restrictions on publicly sharing data—e.g. participant privacy or use of data from a third party—those must be specified.

Reviewer #1: Yes　→Thank you for reviewing the revised manuscript.

Reviewer #2: Yes　→Thank you for reviewing the revised manuscript.

5. Is the manuscript presented in an intelligible fashion and written in standard English?

PLOS ONE does not copyedit accepted manuscripts, so the language in submitted articles must be clear, correct, and unambiguous. Any typographical or grammatical errors should be corrected at revision, so please note any specific errors here.

Reviewer #1: No　→We have corrected the points raised in the point by point response.　 In addition, we have followed the points raised in the first peer review.　We have engaged a professional medical manuscript editing service to revise the manuscript at our own expense. The revised manuscript, addressing these comments, is now submitted. If there are any parts that do not conform to standard English, please provide specific feedback, and we will promptly make corrections.

Certificate of English ProofreadingHonyaku Center Inc. certifies that the manuscript entitled

Investigation of the Effectiveness of No-reference Metric in Image Evaluation in Nuclear Medicine

has been edited and corrected to the highest standards.

Neither the contents of this manuscript nor the author’s intentions have been altered in any way.

This manuscript has been edited and corrected by an experienced proofreader who is a native speaker of

English and who is under the direct supervision of Honyaku Center Inc.

ISSUED ON　March 28, 2024　AUTHORS　Shigeaki Higashiyama　JOB CODE　BQYRQ_2　Yasuo Terashima

Reviewer #2: Yes　→Thank you for reviewing the revised manuscript. We will proceed with the submission as it is.

6. Review Comments to the Author

Please use the space provided to explain your answers to the questions above. You may also include additional comments for the author, including concerns about dual publication, research ethics, or publication ethics. (Please upload your review as an attachment if it exceeds 20,000 characters)

Reviewer #1: (No Response)　 →Thank you for reviewing the revised manuscript. We will proceed with the submission as it is.

Reviewer #2: All the comments have been addressed. The authors modified the paper based on the suggested comment. Iwouls suggest accept this paper.　 →Thank you for reviewing the revised manuscript. We will proceed with the submission as it is.

**Point-by-point response**

・98: 2 6 MBq basis for inclusion.

⇒The Japanese Society of Nuclear Medicine has publicly released a procedure manual for phantom tests. We used the radiation dose from the FDG and Amyloid Brain PET Imaging Phantom Test Procedure Manual (in Japanese). <https://jsnm.org/wp_jsnm/wp-content/uploads/2021/02/Dementia_PhantomTest_20210208.pdf>

・99：PET/CT scannerのBiograph visionは450？600？

・99: The PET/CT scanner used was the Biographic vision 450 or 600?

⇒ We have made changes to lines 86 and 97.

・112：Pixel size 0.825 mm is only correct for a matrix size of 880 when the transverse FOV is 726 mm.

⇒ With an FOV of 363 mm, for a Matrix Size of 440, the voxel size becomes 0.825 mm.

We have added a description of the FOV in the 99th line.

・113: Is 3 mm correct for Slice thickness instead of 3 cm?

⇒ We have made changes to lines 101.

116: Imaging conditions for computed tomography attenuation correction should be stated.

　　The description of Random correction should also be added.

⇒ We have described the reconstruction conditions using CT and the random correction in lines 104 and 111.

・120: 440 matrix size is more appropriate than 440 pixel size.

⇒ We have made changes to lines 109,110.

：What does 0.4125 mm×0.4125×2 mm^３^mean? If the transverse FOV is 726 mm and the matrix size is 440, the pixel size is 1.65 mm; if the matrix size is 880, the pixel size is 0.825 mm. If the magnification factor is 2x, the pixel size is 0.825 mm for a 440 matrix size and 0.4125 for an 880 matrix size.

⇒ The FOV is 363 mm. We have added this to line 100.

・122: Statistical analysis instruments are described, but significance levels are not stated.

⇒ We have added p-value settings to lines 217 and 220.

・235：CV should be assessed on a uniform phantom. There is also poor evidence for the size and location of the ROI; if CV is measured once in each series, it should be measured at least three times to account for measurement error.

⇒ We evaluate with ROIs of 5 mm diameter when conducting clinical image evaluations at our facility. In line with that evaluation method, we conducted evaluations using ROIs of 5 mm diameter. This information has been added to line 226.

⇒ We did not conduct experiments with a pool phantom because we aimed for evaluations based on images intended for clinical examinations.

・394: acquisition time is appropriate, not capturing time.

⇒ We have made changes to lines 381.

・The matrix size and color scale should be added to Fig 1 and Fig 2 respectively.

⇒We have modified lines 566 to 572.

・The names of the vertical axes in Fig 6 should be added.

⇒ We have made corrections to lines 218 to 221 and added them to Fig. 6.

・The names of the vertical and horizontal axes in Figure 8 are mandatory. If the vertical axis is the CV value and the horizontal axis is the acquisition time, they should be described.

⇒We have made modifications to Figure 8.

・Is the value obtained for the 440 and 880 matrix size for 330 s in the CV results in Table 4 correct?

⇒Thank you very much for your detailed feedback. There are no mistakes in the parts you pointed out, and the results are accurate.

・The CV values were similar for the 880 matrix size and the 440 matrix size. However, the visual evaluation results show that the 880 matrix size is better for all acquisition time when compared to the 440 matrix size.

⇒Thank you very much for your detailed feedback. There are no mistakes in the parts you pointed out, and the results are accurate.
