# Supplementary material for: Investigation of the effectiveness of no-reference metric in image evaluation in nuclear medicine
Source: PLoS One. 2024 Nov 21;19(11):e0310305. doi: 10.1371/journal.pone.0310305 (PMC11581283; doi:10.1371/journal.pone.0310305)
Supplement: Attachment — Submitted filename: 20240808Reviewer Comments.docx [file pone.0310305.s006.docx]

Thank you for your careful review and your comments.

We have made changes to comply with your suggestions.

Changed parts are written in red.

**Reviewer Comments**

84：Additional references to the guidelines.

→References have been added and additional information has been added.

100, 112, 113, 115：Half-width space added between number and unit.

→We made the changes as you suggested.

125: 880 matrix size is more correct than 880 pixels. Please check the other text as well; Fig. 1a should be a PET image with 440 matrix size, not 440 s.

→We made the changes as you suggested.

We evaluate with ROIs of 5 mm diameter when conducting clinical image evaluations

at our facility.

⇒ In the diagnostic imaging, it is necessary to state the validity of the 'method of placing a 5 mm ROI on the brain PET image', as shown in Fig. 4. For example, the rationale based on guidelines and previous studies.

→References have been added and additional information has been added.

The number of measurements should be performed at least three times due to the variability of the data.

→　To check the variability of the data, we examined it in three locations: the frontal lobe, the temporal lobe, and the occipital lobe. Changes and additions to the table and figure have been added at lines 226、331 and 387.

Addition of units for acquisition time in Fig. 8.

→We made the changes as you suggested.
