# Supplementary material for: Investigation of the effectiveness of no-reference metric in image evaluation in nuclear medicine
Source: PLoS One. 2024 Nov 21;19(11):e0310305. doi: 10.1371/journal.pone.0310305 (PMC11581283; doi:10.1371/journal.pone.0310305)
Supplement: Attachment — Submitted filename: Reviewer Comments.docx [file pone.0310305.s005.docx]

**Reviewer Comments**

84：Additional references to the guidelines.

100, 112, 113, 115：Half-width space added between number and unit.

125: 880 matrix size is more correct than 880 pixels. Please check the other text as well; Fig. 1a should be a PET image with 440 matrix size, not 440 s.

We evaluate with ROIs of 5 mm diameter when conducting clinical image evaluations

at our facility.

⇒ In the diagnostic imaging, it is necessary to state the validity of the 'method of placing a 5 mm ROI on the brain PET image', as shown in Fig. 4. For example, the rationale based on guidelines and previous studies. The number of measurements should be performed at least three times due to the variability of the data.

Addition of units for acquisition time in Fig. 8.
